# Enhanced Interface Adhesion by Novel Eco-Epoxy Adhesives Based on the Modified Tannic Acid on Al and CFRP Adherends

**DOI:** 10.3390/polym12071541

**Published:** 2020-07-12

**Authors:** Nataša Z. Tomić, Mohamed Nasr Saleh, Sofia Teixeira de Freitas, Andreja Živković, Marija Vuksanović, Johannes A. Poulis, Aleksandar Marinković

**Affiliations:** 1 Innovation center of Faculty of Technology and Metallurgy in Belgrade ltd, Karnegijeva 4, 11120 Belgrade, Serbia; 2 Structural Integrity & Composites Group, Faculty of Aerospace Engineering, Delft University of Technology, 2629 HS Delft, The Netherlands; S.TeixeiraDeFreitas@tudelft.nl (S.T.d.F.), J.A.Poulis@tudelft.nl (J.A.P.); 3 Faculty of Technology and Metallurgy, University of Belgrade, Karnegijeva 4, 11070 Belgrade, Serbia, sd.andreja@yahoo.com (A.Ž.); marinko@tmf.bg.ac.rs (A.M.); 4 “Vinča” Institute of Nuclear Sciences, University of Belgrade, Mike Petrovića Alasa 12-14, 11351 Belgrade, Serbia; mdimitrijevic@tmf.bg.ac.rs

**Keywords:** adhesives, epoxides, tannic acid, bell peel test, adhesion parameter

## Abstract

This paper presents a new process for obtaining eco-epoxide adhesives synthesized from bio-renewable raw material (tannic acid—TA) and used for bonding lightweight materials (aluminum (Al) and carbon fiber reinforced polymer (CFRP)). Two synthesized bio-epoxy components based on TA, (A) glycidyl ether and (B) glycidyl phosphate ester of TA, were used as a replacement for the toxic epoxy component based on Bisphenol A. The effect of eco-epoxy components on the interface adhesion was measured by the determination of adhesion parameter *b*, which was compared to the reference epoxy (REF). The increase of adhesion parameter *b* was 77.5% for A and 151.5% for B. The adhesion of both eco-adhesives was tested using the bell peel test (BPT) with the Al and CFRP adherends. When compared to REF, the average peel load for B was 17.6% (39.3%) and 58.3% (176.9%) higher for the Al and CFRP adherends, respectively. Complete adhesion failure of REF reflected the weak adhesion to both Al and CFRP, which was improved by the addition of eco-epoxy components A and B showing the presence of cohesive failure. The microhardness testing method of interface adhesion was proven to be a fast and reliable testing method, providing a qualitative indication in adhesive selection.

## 1. Introduction

Nowadays, thermosetting polymers have a significant share in plastic production (20%) due to their outstanding properties [1]. Epoxy materials are characterized with high a cross-linking density, their ease of use, and high applicability in the industry. They are essential for structural lightweight materials for aerospace, maritime, and automotive industries, as well as used as adhesives, coating, paints, etc. [2]. Cured epoxy polymer networks possess numerous hydroxyl and benzoyl functional groups that contribute to their high thermal stability, great mechanical properties, and their adhesion ability to different adherends [3]. In addition, epoxides show low thermal shrinkage, resistance to chemicals, moisture, corrosion, and fatigue [4,5].

Bisphenol A (BPA) is a petroleum based chemical that represents a building block of most epoxy materials (>90%). The aromatic core of such compounds contributes to the stability, as well as high thermal and mechanical properties of epoxy materials. Bisphenol A diglycidyl ether (DGEBA) is an epoxy-functionalized component based on BPA, which is also used in the production of food-related and health products such as bottles, containers, dental materials, and products for skin care [6]. However, the BPA leaching during exploitation and waste management represents a threat to human health and the environment [6,7,8]. The activity of the BPA structure in living organisms showed that it belongs to the group of synthetic estrogens [9]. In addition to its side effects on the hormonal balance, BPA is found to be a carcinogen mutagen, reprotoxic, and endocrine disruptor [10]. Therefore, the presence of BPA is banned in baby bottles, and its usage in food-related/health products is strictly controlled. Toxicity and the non-recyclability of BPA-based epoxides led to the increased efforts of both academia and industries to find an appropriate eco-friendly replacement [11,12,13]. Thus, extensive studies have been conducted in order to investigate different bio-based resources for the production of BPA component counterparts, but only few of them were commercialized. Eco-epoxy resins, currently available in the market, are based on epoxidized vegetable oils [14,15] and cardanol [16]. Vegetable oils have long aliphatic chains whose unsaturated bonds are converted to low reactivity epoxy groups [1]. This structure is preventing them from achieving high glass transition temperatures and mechanical properties, and thus replacing conventional BPA-based materials [17].

Efforts have been made in adhesive technology to use bio-renewable sources in producing commercial (petroleum/based) counterparts with equal or even better performance. Abundant bio-based raw materials like lignin, tannin, and cellulose consist of numerous hydroxyl groups, increasing their reactivity, which is favorable in the production of adhesives [18,19,20]. In addition, the high content of phenolic groups contributes to higher fire resistance of the system. With the aim of replacing the conventional BPA component in epoxy resins, reactive epoxy functional groups are introduced into the structure to establish bio-based epoxy networks [21,22,23]. Some tannins (i.e., from plants such as quebracho and wattle) are used in the production of formaldehyde wood adhesives since the 1970s [24]. To reduce formaldehyde emission, a new formaldehyde-free system was created due to the presence of a catechol group in tannin that reacted with polyethyleneimine (PEI) with strong adhesion and water resistivity [25]. Further studies on the modification, characterization, and wide-spread use of tannins in different adhesive systems, not only for wood application, would gain higher benefit of high importance for the commercialization by the stakeholders.

The aim of this study is to investigate the interface adhesion of novel eco-epoxy adhesives by the addition of two types of modified tannic acid: (A) glycidyl ether and (B) glycidyl phosphate ester of TA, which are used as a bio-based replacement of the BPA-based epoxy component. The majority of structural epoxy adhesives, used in aerospace, contain a BPA component, and thus the adhesion effects were analyzed on two different substrates: aluminum (Al) and carbon fiber reinforced polymer (CFRP), which are used for lightweight structures. Methods used for characterization were the microhardness testing method, the bell peel test (BPT), and microstructural analysis of fractured surfaces. In addition, proving that the microhardness testing method of the interface adhesion is a reliable and fast testing method will enable its use as qualitative indicator in adhesive selection.

## 2. Materials and Methods

### 2.1. Adhesives and Adherends

The chemicals used in the synthesis of modified tannic acid (TA)—epichlorohydrin (EPH), sodium hydroxide (NaOH), deionized water (MiliQ), chloroform, *N*-methyl-2-pyrrolidone (NMP), tetrahydrofuran (THF), phosphorus oxychloride (POCl_3_), glycidol, magnesium sulfate, calcium chloride—were used as received and supplied from Sigma-Aldrich Chemie Gmbh, Steinheim, Germany. The solvent used for the surface cleaning, acetone, was supplied from Sigma Aldrich, USA. The reference adhesive was selected to be a BPA-based epoxy (LG700 epoxy component and HG 700R curing agent) and was supplied from GI-NI ltd., Belgrade, Serbia (epoxy value 0.62, *T*_g_ = 79.4 °C).

Two types of substrates were selected: aluminum alloy 2024 and CFRP composites HexPly 8552 unidirectional prepreg epoxy resin in combination with AS4 carbon fiber (Hexcel Composites, Cambridge, UK). CFRP laminates were manufactured in the autoclave with a curing cycle of 180 °C for 120 min at 7 bars pressure. During manufacturing, the surface of the laminates was in contact with a fluorinated ethylene propylene copolymer release film (FEP Copolymer A 4000 clear red, Airtech Europe, Niederkorn, Luxembourg).

Aluminum and CFRP plates were cut into 5 replicates according to the standards for the bell peel test (BPT), 150 × 300 mm each [26]. Each sample was 25 mm wide. However, the thickness of the Al plate samples for BPT/Al was 1.6 mm for the rigid and 0.6 mm for the flexible adherend and for BPT/CFRP 2.4 mm for the rigid ([0°/90°]_6_) and 0.5 mm for the flexible adherend [0°/90°].

#### 2.1.1. Synthesis of Glycidyl Ether of TA

Glycidyl ether of TA was obtained via the reaction of TA and EPH at 80 °C and at 1:1.5 wt. ratio of TA to NaOH [27]. EPH (15 g) was dissolved in THF (15 mL) at room temperature under magnetic stirring for 30 min. Subsequently, the solution was placed in a 100 mL three-neck round-bottomed flask equipped with a reflux condenser, pressure equalizing dropping funnel, and nitrogen inlet tube. Then, the TA (3 g) was added to the solution and heated up to 80 °C under magnetic stirring. Afterwards, 22.5 mL of 20% NaOH solution (4.5 g NaOH in 18 mL of water) was added dropwise using a dropping funnel while stirring. The reaction lasted for 3 h at 80 °C. The obtained solution was left to cool down, and then was slowly added to 200 mL of cold MiliQ water. The extraction of the product with toluene (3 × 70 mL) was followed by drying with MgSO_4_ overnight. The toluene solution was filtrated and transferred to a flask equipped with a short distillation column, Liebig condenser, and recipient fitted well to sustain high vacuum (~1 kPa). Concomitant increase of temperature (2 °C/min) was followed by pressure decrease in order to remove all volatile residues in glycidyl ether of TA. The obtained product was a highly viscous brownish liquid.

#### 2.1.2. Synthesis of Glycidyl Phosphate Ester of TA

Glycidyl phosphate ester of TA was obtained according to a novel developed procedure for obtaining fire retardant epoxy components [28]. At room temperature, TA (6 g) was dissolved in 50 mL of a 1:1 ratio mixture of chloroform and NMP in a 250 mL three-neck round-bottomed flask equipped with a vacuum distillation apparatus and two pressure equalizing dropping funnel. After 30 min, the temperature of the oil bath was set to 70 °C. One dropping funnel was filled with a solution of 9.75 g of POCl_3_ in 20 mL of chloroform, and the second one with 9.42 g of glycidol dissolved in 40 mL of chloroform. As soon as the reaction temperature reached 70 °C, the reaction was initiated by a dropwise addition of POCl_3_ solution under constant stirring for 1 min and a low vacuum (~1 kPa). After 10 min, glycidol was added in a dropwise manner for 2 min. Using the same time intervals (1 and 2 min), the addition was continued until the whole amount of reactants was reached. Then, the temperature was set to 85 °C with the vacuum gradually increasing, until all the chloroform was removed. The reaction was completed after 12 h and then the vacuum was increased (10 Pa) in order to remove NMP. When the reaction was finished, the product purification was performed analogously to glycidyl ether of TA. The obtained product was a highly viscous brown liquid.

**Scheme 1:**
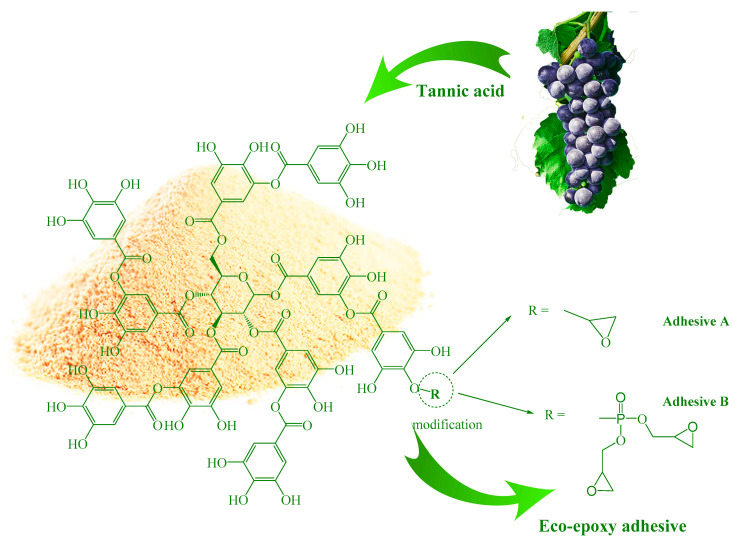
Chemical structure of modified tannic acid for the aim of obtaining eco-epoxy components.

The chemical structure of both types of adhesive components obtained by the modification of TA are presented in Scheme 1.

### 2.2. Surface Treatment

**Figure 1:**
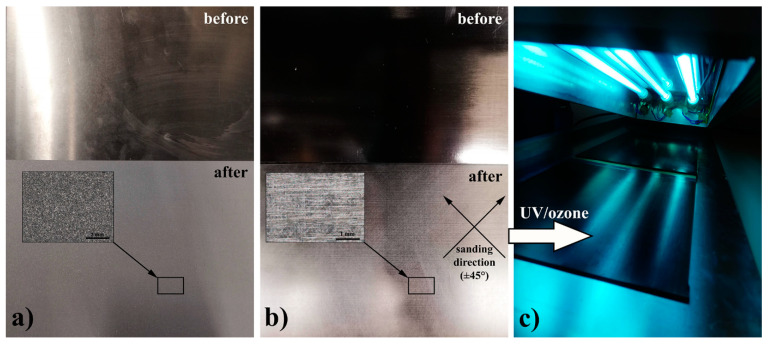
Surface appearance before (upper image) and after (lower image) surface treatment for (**a**) aluminum (Al), (**b**) carbon fiber reinforced polymer (CFRP), and (**c**) UV/ozone treatment for surface treatment of CFRP.

Prior to bonding, the surface preparation of the aluminum samples was as follows: I—acetone cleaning, II—grit blasting with Al_2_O_3_ powder (Corublast Super Z-EW No. 40, Ø 0.35–0.50 mm, Figure 1a), III—acetone cleaning, and IV—air blow duster gun.

For the CFRP case, the surface is prepared in the following steps: I—acetone cleaning, II—sanding (240 grit size) in ±45° direction (Figure 1b), III—acetone cleaning, IV—air blow duster gun, and V—UV/ozone treatment (7 min of exposure, Figure 1c).

Figure 1c shows the UV/ozone apparatus used for the surface treatment of CFRP samples that consists of three UV lamps (30 W, λ = 184.9 nm and 253.7 nm) with a sleeve of natural Quartz (UV-Technik, Wümbach, Germany) at atmospheric conditions. The UV/ozone treatment is an easy to use dry cleaning/surface activation method using UV light in an ozone atmosphere (generated from the environmental air) [29]. During the UV/ozone treatment, the contaminants or their free radicals generated by photolysis react with atomic oxygen forming volatile molecules such as CO_2_, H_2_O, N_2_, and O_2_ [30,31]. However, before performing the treatment, it is necessary to perform preliminary bulk cleaning steps such as mechanical (sanding) and chemical (detergents and/or solvents, e.g., acetone). Besides the contaminant removal, UV/ozone treatment is employed for the oxidation of the CFRP surface and the introduction of hydroxyl and carboxylic groups that would improve the interfacial adhesion [31]. Exposure times higher than 5 min were found to be successful in the introduction of a significant amount of oxygen containing functional groups in cured epoxy in printed circuit boards [32,33,34,35]. In addition, a significant increase in surface roughness was found due to the etching effect of UV/ozone treatment of epoxides [32].

**Figure 2:**
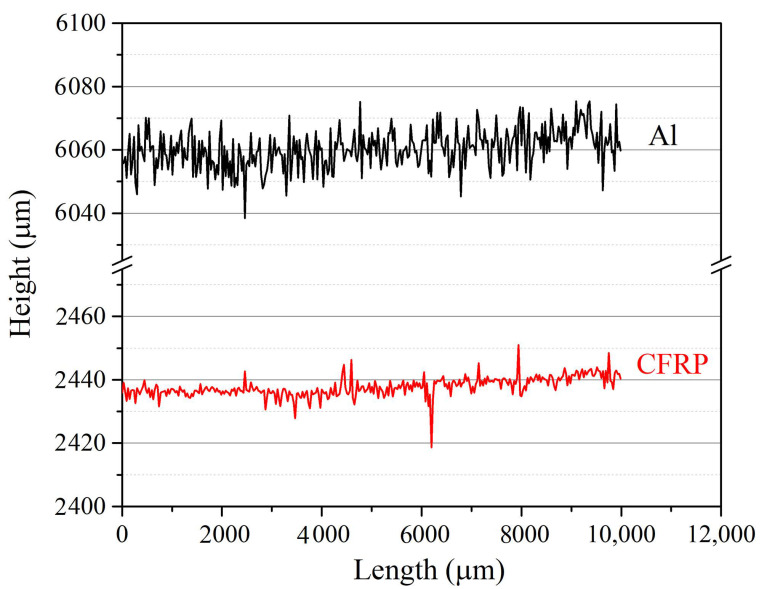
Line profile of both adherends, Al and CFRP.

The surface roughness parameters are analyzed as arithmetical mean height (Ra, Pa, Wa) and as maximum height profile (Rz, Pz, Wz) [36]. Results are shown in Table 1. In addition to the visual differences between the Al and CFRP surfaces seen in Figure 1a,b, the determined surface roughness parameters shed some light on the influence of the type of surface treatments performed. The ratio of the arithmetical parameters (Ra/Pa/Wa) indicated 74% higher surface roughness of Al compared to CFRP, but the maximal values (Rz/Pz/Wz) showed ~200% higher roughness of CFRP. These results indicated that sanding of CFRP surface did not provide uniform surface morphology as obtained after grit blasting of Al. Line profiles of surface roughness are determined in order to obtain a detailed insight into the surface roughness of the treated materials, as shown in Figure 2. The Al surface showed higher frequency of surface height peaks, indicating uniform abrasion of the surface by sand blasting, seen as higher arithmetical parameters than for CFRP (Table 1). Solitary height peaks noticed in the profile line for CFRP originated from surface abrasion by sand paper, which are suggested by Rz/Pz/Wz parameters. These obtained data about the surface roughness due to the different pretreatments may suggest a better mechanical interlocking in the case of Al as opposed to CFRP.

### 2.3. Bonding of Al–Al and CFRP–CFRP

Three adhesives were selected for testing the adhesion effects on both of substrates: (1) reference epoxy adhesive (REF); (2) epoxy adhesive with 15 wt.% of glycidyl ether of tannic acid (adhesive A); and (3) epoxy adhesive with 15 wt.% of glycidyl phosphate ester of TA, (adhesive B). Glass bead spacers (150–250 µm, Sigma Aldrich Chemie Gmbh, Steinheim, Germany), used for the adhesion thickness control, were mixed with the adhesives at 0.1 wt.% prior to bonding. Final thickness of samples was 210 µm ± 40 µm. The curing of the specimens was performed at room temperature for 24 h, with post-curing at 70 °C for 4 h. After curing, 25 mm wide samples were cut.

## 3. Experimental procedure

### 3.1. Characterization Methods

#### 3.1.1. FTIR Analysis

FTIR spectroscopy of epoxy components used in the adhesive preparation was performed in order to confirm the obtained structure of the synthesized components and to compare the presence of the functional groups that can affect the bonding interface. The analysis was done using a Nicolet^TM^ iS^TM^10 spectrometer (Thermo Fisher Scientific Inc, Waltham, Massachusetts, USA). The attenuated total reflectance (ATR) sampling technique was used, which is equipped with a DTGS detector and Golden Gate system with a diamond crystal. The OMNIC software was used to record spectra of samples in a wavelength range of 4000–500 cm^−1^ with a 4 cm^−1^ resolution. Afterwards, they were post-processed with ATR correction.

#### 3.1.2. Contact Angle Measurement

The adhesive affinity to both adherends, Al and CFRP, was assessed by measuring the wetting angle. A drop of the adhesive mixtures was placed on the aluminum adherend after the surface treatment. Five wetting angle measurements were performed using the same test conditions. The images of the samples were taken with an optical microscope (Smart 5MP Pro, Delta Optical Instruments, Inc, North Little Rock, Arkansas, USA), and the contact angles were determined using the image analysis software Image-Pro Plus 4.0 (Media Cybernetics Inc, Rockville, Maryland, USA).

#### 3.1.3. Adhesion Parameter *b*

The microhardness of the adhesives on aluminum surfaces was characterized by the micro Vickers hardness (HV) tester Leitz, Kleinharteprufer DURIMETI, using an original quadrangular pyramid diamond indenter with an angle of 136° [37]. In order to obtain HV values for the use in the Chen–Gao mathematical model [38], the tests were performed using a range of loads, i.e., 15, 25, 50, 100, 200, 300, and 500 gf. Microhardness indentation was performed at room temperature with three measurements using the same test conditions as per ASTM E384-16 [39]. An image analysis software, Image-Pro Plus 4.0 (Media Cybernetics), was utilized to determine the values of the diagonals of the microhardness indents captured by the optical microscope, Carl Zeiss – Jena, NU2, 73447 Oberkochen, Germany. The micro Vickers hardness number (VHN) is calculated according to Equation (1):

(1) $$VHN=2\frac{\cos\left(22{}^{\circ}P\right)}{d^{2}}=1.8544\frac{P}{d^{2}},$$

where *P* (kgf) is the applied load and *d* (mm) is the average length of the indentation diagonals [37].

#### 3.1.4. Bell Peel Test (BPT)

**Figure 3:**
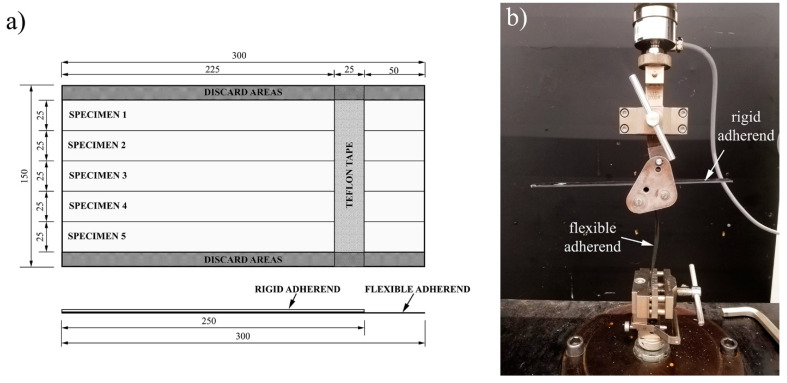
Bell peel test (BPT): (**a**) preparation of samples and (**b**) experimental set-up.

BPT was performed with the aim to study the adhesion effect of the synthesized eco-epoxy components on both adherends, Al and CFRP. The experimental procedure for BPT was based on the standard test method described in ASTM D3167 [26]. Figure 3a shows a typical BPT samples sketch with flexible and rigid adherends. Figure 3b depicts the test set up. Testing was carried out using an electromechanical Zwick Roell machine (Zwick, Ulm, Germany) with a load cell capacity of 10 kN. The testing speed was 125 mm/min. A total of five specimens were tested under each test condition. During testing, the load and the crosshead displacement were recorded every 0.1 s and 0.1 mm, respectively.

#### 3.1.5. Fractured Surface and Roughness Characterization

The post-mortem fractured surface of representative samples from each tested group was analyzed in order to determine the type of adhesion failure using 3D optical microscope with a wide-area 3D measurement system, type VR-5200 from Keyence, Itasca, Illinois, USA. The surface roughness parameters (Ra/Pa/Wa and Rz/Pz/Wz) and the corresponding statistical analysis were carried out by the VR-5000 Series Analyzer Software.

## 4. Results and Discussion

### 4.1. FTIR Analysis

**Figure 4:**
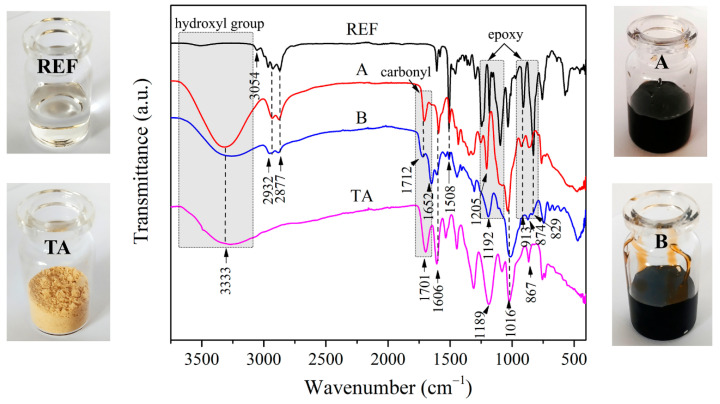
FTIR spectra of used epoxy components for adhesives reference epoxy (REF), glycidyl ether (A) and glycidyl phosphate ester (B) of tannic acid, and neat tannic acid (TA) and their appearance.

FTIR analysis of the modified TA and neat TA was performed to follow the structural changes in the course of modification and to analyze the differences in the content of the functional groups that can affect the adhesive and cohesive properties of the obtained adhesives (Figure 4). The characteristic structural changes of TA by the epoxidation process is reflected by the existence of C–H vibrations at 2932 and 2877 cm^−1^ for both A and B when compared to TA. Other characteristic changes were observed as deformation vibration of epoxy C–O group at 913 cm^−1^ and epoxy ring C–O–C stretching vibration at 829 cm^−1^ [40]. Component B showed the shift in vibration from 1189 to 1192 cm^−1^ and from 867 to 874 cm^−1^ due to the interference of phosphate and the epoxy group. This is the most significant indicator of the obtained glycidyl phosphate ester of TA. In addition, the carbonyl group vibration at ~1700 cm^−1^ was noticed for the TA-derived components due to the ester linkage of the TA core. The REF epoxy component showed characteristic peaks of diglycidyl ether of Bisphenol A (DGEBA): C–O deformation band centered at 915 cm^−1^, C–H stretching of the terminal epoxy at 3054 cm^−1^, broad O–H stretching vibration at ~3500 cm^−1^, bands of ether linkage at 1000–1100 cm^−1^, oxirane C–O–C stretching at 829 cm^−1^, oxirane C–O stretching at 913 cm^−1^, stretching of C–O–C ethers at 1034 cm^−1^, aromatic C–C stretch at 1508 cm^−1^, aromatic ring stretching at 1609 cm^−1^, aliphatic C–H, CH_2_, and C–H aromatic stretch at 2965–2877 cm^−1^, oxirane ring C–H stretching at 3054 cm^−1^ [41].

Based on the presented FTIR spectra, it is clear that the obtained A and B components showed higher polarity due to the presence of hydroxyl groups which can improve the adhesion to both Al and CFRP by establishing hydrogen bonding, and Van der Waals intermolecular forces with the carbonyl group. On the other hand, REF components showed a higher amount of epoxy groups increasing the cross-linking density, and thus leading to a more brittle material.

### 4.2. Contact Angle

Wetting theory suggests that the adhesion is a result of the molecular contact between the substrate-adhesive and the forces developed. The adhesive should have a lower surface tension than a critical surface tension of a substrate in order to establish good wetting of a solid surface [42]. Van der Waals forces are considered weak to establish the adhesion strength, but still can contribute to the overall adhesion. Hydrogen, covalent, and ionic bonding between the adhesive and the adherends represent the main mechanisms in the formation of adhesion bonds with a surface by chemical forces. The wetting angle is influenced by two types of interactions: (a) interactions between the polymer chains of the adhesive contributing to the cohesive strength and (b) interfacial interactions between the adhesive and the adherend, contributing to the adhesion strength [43]. Table 2 presents the values of the wetting angles of the adhesives on both adherends, Al and CFRP. An increase of the wetting angle for adhesives A and B on the CFRP adherend in comparison with REF was noticed. This suggested the presence of a higher amount of intermolecular interactions in the adhesive itself due to the establishment of stronger hydrogen bonding by the introduction of the modified TA components. The obtained results suggest slightly better affinity/compatibility of the REF adhesive with CFRP than Al due to its epoxy nature. The compatibility with Al surface was increased (lower wetting angle) for adhesive A due to the effective hydrogen bonding between the TA component and the free surface hydroxyl groups of the oxidized Al_2_O_3_ layer. A higher wetting angle of adhesive B relative to A, for both adherends, can be attributed to the increased amount of interaction by hydrogen bonding transfer of the phosphate group. Phosphorus is acting as a ligand atom transferring hydrogen bonding from a hydrogen bond donor (or antecedent atom of a hydrogen bond donor) via oxygen as atom of a ligand [44]. In the case of CFRP, the introduction of polar groups for adhesive A was not that favorable as in the case of Al, and it did not cause significant changes in wetting angle. The wetting angle of adhesive B showed the highest value when compared to the REF adhesive, i.e., 27.6%. The values of the wetting angle for all used adhesives/adherends were in the range of 29.3–37.4°, which indicated high wetting and affinity to Al and CFRP surfaces [45].

### 4.3. Adhesion Parameter b (Microhardness Model)

The adhesion parameter *b* is determined as the ratio of the plastic zone radius formed under a micro-indenter and the depth of indentation, which is influenced by the applied film (adhesive), the adherend, and the established adhesion [46]. If a weak adhesion is established, then the strain discontinuity across the interface is allowed, but the strong adhesion of the interface causes distortion of the plastic zone. These main differences enable the determination of the adhesion quality by distinguishing and measuring the indent geometry [38]. A model on the CFRP was not possible to obtain since the model is applicable only for soft films on rigid substrates or rigid films on soft substrates (the difference in rigidity between the adhesive and CFRP is not significant).

**Figure 5:**
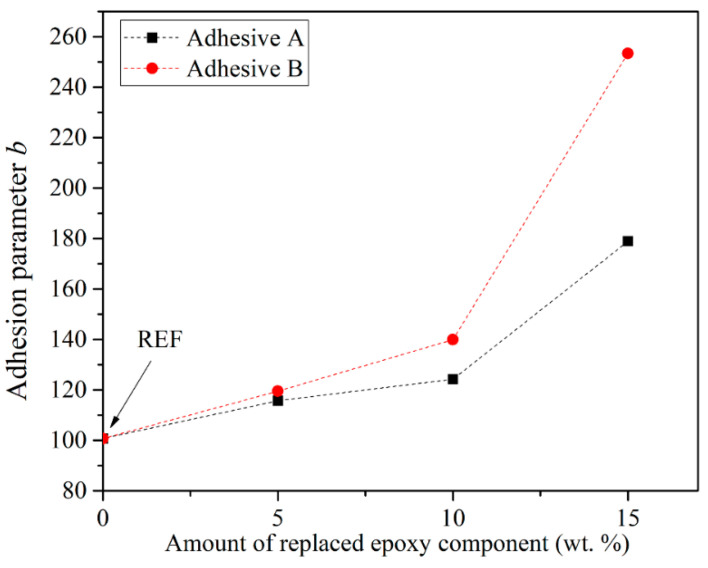
Adhesion parameter *b* determined using the Chen–Gao model [38].

Figure 5 shows the values of the adhesion parameter *b* as a function of the replaced epoxy BPA component. An increase of the adhesion parameter *b* with the increase of TA content indicated an increase of adhesion with Al adherend. This phenomenon can be interpreted in the light of the establishment of a stronger hydrogen bonding on the interface with the introduction of a higher content of TA component. The hydrogen bonding transfer of the phosphate group, observed in the wetting angle measurements, affected the higher rate of the adhesion parameter *b* increase for adhesive B. Higher amounts of TA component in adhesives deteriorated the processability/applicability of the adhesives. Thus, the content of 15 wt.% was selected for BPT. The adhesion parameter *b*, for adhesive B with 15 wt.% of replacement, was 41.6% higher than for adhesive A and 153.4% higher than the REF. These results indicate the strong interface adhesion of adhesive B and its significant improvement when compared to the reference BPA-based epoxy component (REF).

### 4.4. Bell Peel Test (BPT) and Fractured Morphology

BPT was used to obtain an insight into the adhesion failure mechanisms and the peel strength of the used adhesives. The average (*F*_ave_), maximal peel strength (*F*_max_), and failure modes are given in Table 3. Two types of failure mechanisms were observed: adhesive failure (AF) and cohesive failure (CF). The percentage of each failure mode was determined using an image analysis tool, Image-Pro Plus 4.0 (Media Cybernetics). *F*_ave_ and *F*_max_ showed the same tendency for both adherends, Al and CFRP, for the tested adhesives. *F*_ave_ (*F*_max_) of adhesive A was as low as 35.3% (24.4%) and 62.5% (61.5%) on the Al and the CFRP adherends when compared to REF, respectively. *F*_ave_ of adhesive B was as high as 17.6% (39.3%) and 58.3% (176.9%) on the Al and the CFRP adherends when compared to REF, respectively. Regarding the failure mode, REF showed complete adhesive failure for both Al and CFRP indicating weak adhesion. Adhesive A showed a small amount of cohesive failure (residual adhesive parts on both adherends surface). This percentage of CF was the highest for adhesive B on both Al and CFRP in comparison with A and REF adhesives.

It should be noted that, as seen in Figure 3b, the adhesion/cohesion strength of the adhesives used was not sufficient to create the peel angle during testing described in the standard ASTM D3167 [26]. Therefore, the peel load presented in this paper can only be used as a qualitative comparison between the adhesives and adherends used.

**Scheme 2:**
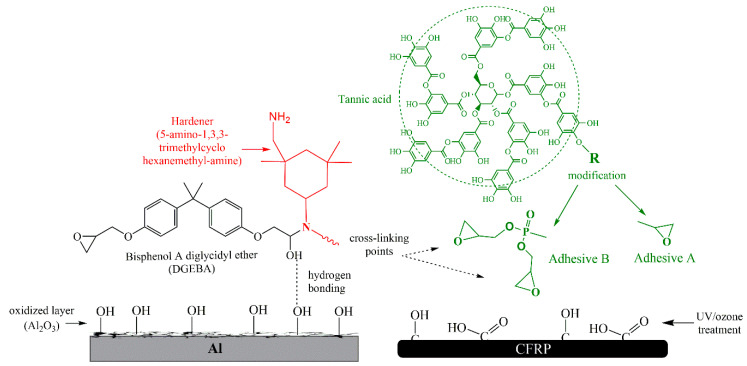
Adhesive–adherend interaction for both Al and CFRP.

The BPT data are influenced by the competition between the cohesive and adhesive forces. The ruling intermolecular forces in the interface and in the adhesives themselves are presented in Scheme 2. With the introduction of the glycidyl ether of the tannic acid (adhesive A), the amount of hydroxyl groups increases. These are responsible for establishing hydrogen bonding with the adherends. On the other side, one epoxy functional group, present in the A component, does not have the ability to cross-link with the epoxy resin. Adhesive A, thus, terminates the epoxy polymer chains by the pendant TA group, reducing the cross-linking density. Consequently, the cohesive strength of adhesive A was significantly reduced which resulted in lower peel loads. However, the interface interactions resulted in minor cohesive failure. On the contrary, the second epoxy group via the phosphoryl group, which enabled effective cross-linking of the adhesive itself, enhanced the cohesive strength of adhesive B. Accordingly, higher peel loads of adhesive B were the result of the improved cohesive and adhesive strength, also causing a higher CF extent. Low intensity Van der Waals forces established between the carbonyl and carboxyl surface groups also contributed in the interface adhesion of CFRP adherends. Beside surface functionalities, the interface strength of both adherends is additionally influenced by the surface roughness, as shown in Table 1. It can be noted that the average arithmetical roughness parameter of CFRP was 43% lower than Al.

**Figure 6:**
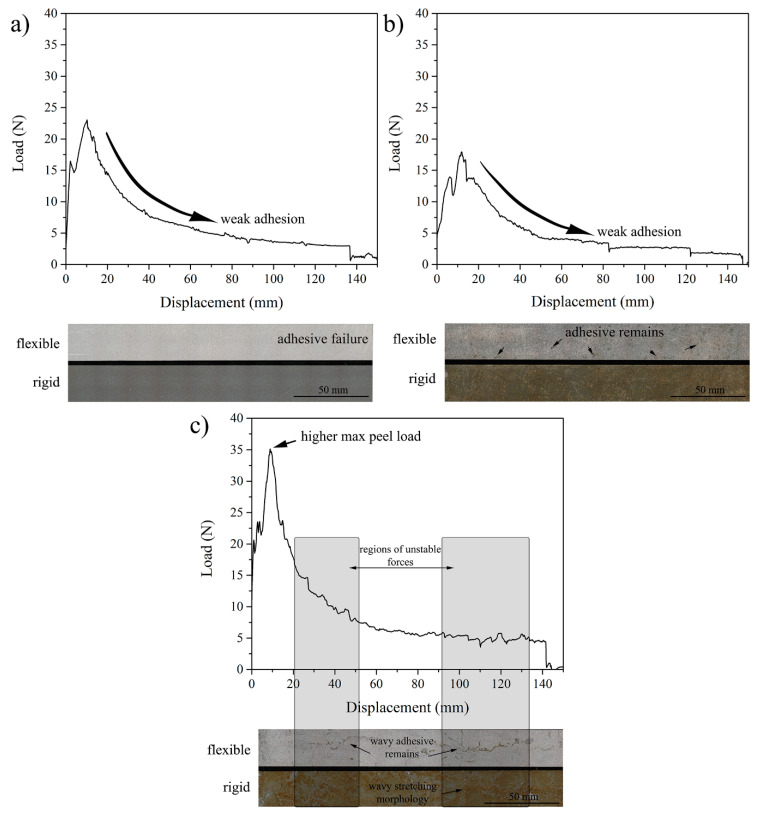
Examples of load–displacement graphs of the bell peel test (BPT) on Al for adhesives (**a**) REF, (**b**) A, and (**c**) B.

Comparison of the load–displacement (L–D) curves with the corresponding fractured Al surfaces after BPT for all the tested adhesives are presented in Figure 6 with the aim of better understanding the relation and effect of the failure mechanism on the peel loads. The weak adhesion strength of REF could be observed by the clean surface of the Al flexible adherend and the continuous load drop as a function of the displacement (Figure 6a). Smooth force decrements for both REF and A showed no significant resistance to peeling. A consequence of the increase in the peel load of adhesive B was the higher percentage of CF than for REF and A. The higher extent of CF commonly leads to higher peel loads than the AF [47]. However, for adhesive A, the increase in CF was not sufficient to have a significant impact in the L–D curves (Figure 6b). The cohesive strength was in a range of the adhesive strength for adhesive A, which caused tearing of its parts that remained of flexible Al surface, increasing the CF content. Adhesive B showed slightly different fracture behavior (Figure 6c). Two regions of unstable forces can be noticed, which are related to the higher percentage of CF and to the crack, jumping from the interface to within the adhesive and vice-versa.

**Figure 7:**
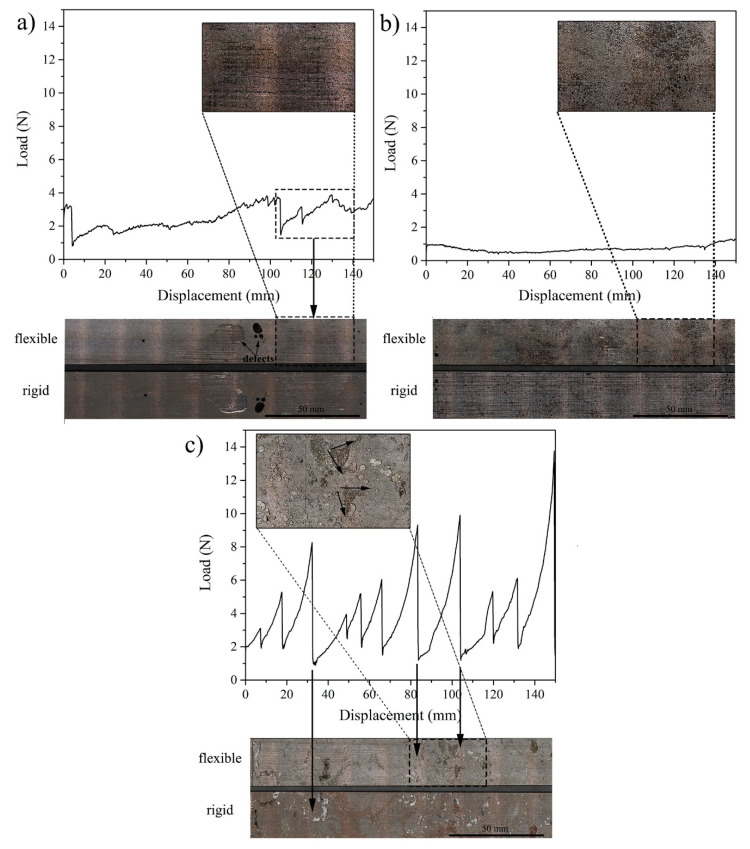
Examples of load–displacement graphs of BPT on CFRP for adhesives (**a**) REF, (**b**) A, and (**c**) B.

Figure 7 shows the load–displacement graphs for the CFRP adherend. Lower peel loads obtained for CFRP-bonded joints, compared to their Al counterparts, were due to the different type of material and thickness of the flexible adherend [47]. Complete AF is once again noticed for REF, but with the appearance of AF ’stripes’ (enlarged part of Figure 7a) corresponding to the sudden load drops, which is indicated with an arrow. Small amounts of adhesive residues (CF) were not enough for creating an increase in the peel load (Figure 7b). Once again, adhesive B exhibited different fracture behavior with significant peel load peaks and drops (Figure 7c). Peel loads are increasing in the areas of CF and suddenly drop at the point of transition to AF, as indicated by arrows in Figure 7c. Maximal peel loads of B were 177% higher than in the case of REF. The fractured surface of B failure showed rough morphology that corresponds to fast damage growth and crack bifurcation (zoom of Figure 7c) [48].

**Figure 8:**
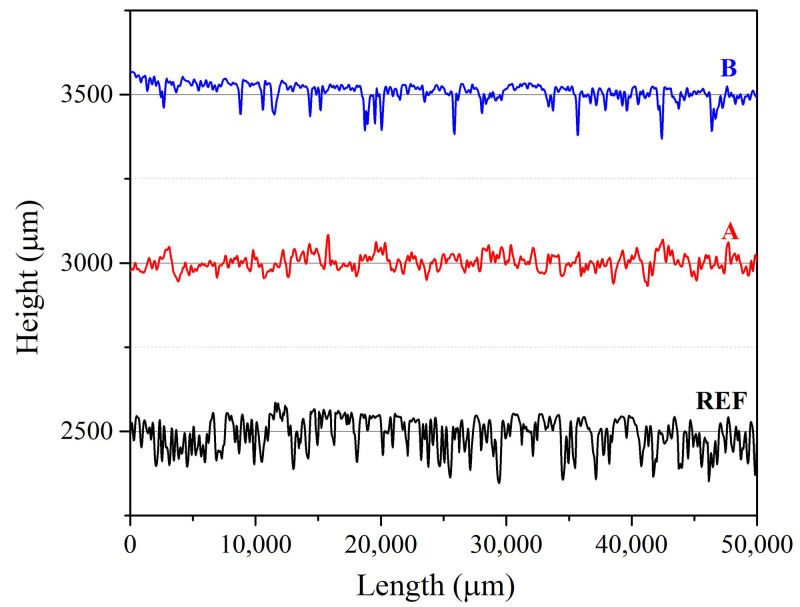
Line profiles of rigid fractured Al surfaces after BPT for adhesives REF, A, and B.

The surface roughness characterization was done on the rigid adherend, after testing, to further analyze the features of the fractured Al surface (Table 4). In the case of AF, a crack between the flexible and rigid adherend occurs, which means that the surface of the flexible adherend remains clean. Thus, the surface of the rigid adherend was the one of interest in order to investigate the roughness caused by CF, defects, and plastic deformation of the adhesives. Figure 8 shows the line profiles of the fractured surfaces, which were analyzed together with the obtained surface roughness parameters. Higher roughness according to Ra/Pa/Wa, Rz/Pz/Wz, and the line profile was found for the REF sample. This high roughness of REF can be attributed to the presence of defects in the adhesive, mapping the morphology of the flexible adherent, and some extent of the plastic deformation of the adhesive. According to the manufacturer specification, the elongation of REF is ~8%. Significantly lower average arithmetical roughness parameters (Ra/Pa/Wa) of adhesive A (52%) as opposed to REF indicate CF on the flexible adherend which remained on its surface, filling peaks/valleys made by grit blasting. The increased value of Ra/Pa/Wa of B when compared to A (57%) was due to the higher extent of CF, while Rz/Pz/Wz values, which are even higher than adhesive thickness, indicate the presence of a higher share of plastic deformation due to the increased ductility of adhesive B (adhesive thickness was 210 ± 40 µm).

### 4.5. Comparison of Adhesion Parameter b and Adhesion Failure

**Figure 9:**
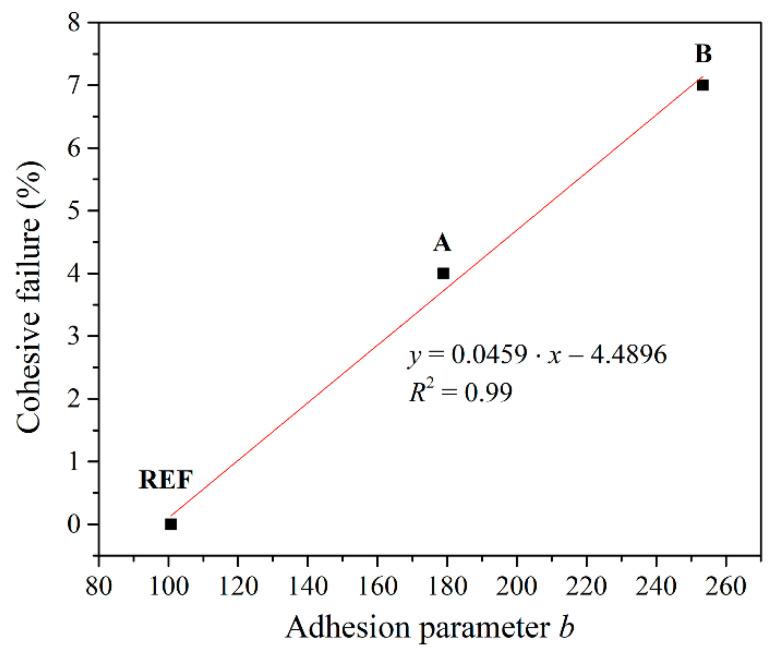
Correlation between the cohesive failure and the adhesion parameter *b* for adhesives REF, A, and B on Al adherends.

Since the adhesion parameter *b* gives an indication of the interface strength, it can be compared and correlated to the amount of cohesive failure on the flexible adherend after BPT. Cohesive failure suggests that the adhesion interface forces prevailed over the cohesive forces and may be an indicator of the adhesion strength, similar to the adhesion parameter *b*. Figure 9 shows a correlation with linear fit (*R*^2^ = 0.99) fitted between the cohesive failure and the adhesion parameter *b*. The obtained results indicated that the fast and easy method for assessment of the adhesion quality, using the adhesion parameter *b*, may be used to predict the differences between the selected adhesives for the same adherend material. Nevertheless, the adhesion parameter *b* cannot predict the differences in peel loads, and its use might be limited only for the preselection of adhesives for specific use.

## 5. Conclusions

Two eco-epoxide components based on TA were synthesized: (A) epoxy functionalized and (B) epoxy ester phosphate derivate of TA, and used as a replacement of the BPA-based component. The effect of the synthesized bio components on the interface adhesion was tested on Al and CFRP, which are commonly used for lightweight structures, and compared to the reference BPA-based epoxide. Adhesion properties were evaluated by contact angle, adhesion parameter *b*, bell peel test (BPT), and fractured surface analysis. This study showed that the introduction of TA-based components contributed to the increase of hydrogen bonding, which further enhanced the interface adhesion. Improvement of the interface adhesion was noticed by the increase in the adhesion parameter *b*, which was 77.5% higher for component A and 151.5% higher for component B. The higher contact angle of component B compared to the one of A was due to the presence of a higher extent of cohesive forces in the adhesive itself. The highest peel loads of B were influenced by both adhesive and cohesive strength. The hydrogen bonding, as a main adhesion mechanism, enhanced the adhesion strength of adhesive B. Moreover, the cohesion strength of adhesive B is improved by higher cross-linking density than adhesive A. Linear correlation between the cohesive failure and the adhesion parameter *b* indicated that the microhardness testing method of interface adhesion can be used as a fast and reliable testing method in the adhesive selection process. The synthesized epoxy ester phosphate derivate of TA (adhesive B) showed enhanced interfacial adhesion on both Al and CFRP, and their high potential as a replacement of the BPA component (DGEBA) was emphasized. Industrial application of obtained eco-epoxy adhesives might consider bonding of secondary/non-structural elements of lightweight structures.

## Figures and Tables

**Table 1:** Results of adherends surface roughness.

	Al		CFRP	
	Ra/Pa/Wa	Rz/Pz/Wz	Ra/Pa/Wa	Rz/Pz/Wz
	µm	µm	µm	µm
Average	4.85	39.75	2.78	119.70
Max	5.22	51.65	3.09	243.34
Min	4.46	33.34	2.47	32.62
Std. dev.	0.27	4.70	0.20	63.23

**Table 2:** Wetting angle values of the used adhesive for Al and CFRP.

Sample	Al	CFRP
REF	34.8 ± 2.2	29.3 ± 3.0
Adhesive A	30.4 ± 3.4	30.5 ± 1.0
Adhesive B	34.3 ± 2.3	37.4 ± 2.6

**Table 3:** Summarized data on average peel loads, maximum peel loads, and failure mechanisms.

	Al				CFRP			
Adhesive ↓	*F*_ave_ (N/25 mm)	*F*_max_ (N/25 mm)	Failure Mode		*F*_ave_ (N/25 mm)	*F*_max_ (N/25 mm)	Failure Mode	
			AF ^b^ (%)	CF ^c^ (%)			AF (%)	CF (%)
REF	3.4 (0.1) ^a^	13.5 (2.3)	100	0	2.4 (0.3)	3.9 (0.5)	100	0
A	2.2 (0.3)	10.2 (1.7)	96	4	0.9 (0.3)	1.5 (0.4)	97	3
B	4.0 (1.2)	18.8 (2.0)	93	7	3.8 (0.5)	10.8 (3.2)	91	9

^a^ Values in parenthesis represent standard variation values, ^b^ AF—adhesive failure, ^c^ CF—cohesive failure.

**Table 4:** Surface roughness of the fractured Al surface after BPT.

	REF		A		B	
	Ra/Pa/Wa	Rz/Pz/Wz	Ra/Pa/Wa	Rz/Pz/Wz	Ra/Pa/Wa	Rz/Pz/Wz
	µm	µm	µm	µm	µm	µm
Average	59.30	282.84	28.48	223.24	44.71	326.03
Max	72.21	296.13	36.25	306.78	53.23	348.74
Min	53.31	270.46	21.35	171.05	36.47	318.36
Std. dev.	4.82	8.76	4.46	38.36	5.59	8.04
